# A Comparison of a Standard Macintosh Blade Laryngoscope, Pentax-AWS Videolaryngoscope and Intubrite Videolaryngoscope for Tracheal Intubation in Manikins in Sitting and Prone Positions: A Randomized Cross-Over Study

**DOI:** 10.3390/diagnostics10080603

**Published:** 2020-08-18

**Authors:** Tomasz M. Gaszyński

**Affiliations:** Department of Anesthesiology and Intensive Therapy, Medical University of Lodz, 90-419 Lodz, Poland; tomasz.gaszynski@umed.lodz.pl

**Keywords:** airway management, intubation, videolaryngoscopes, position for intubation

## Abstract

Background: Intubation of a patient in different positions may be done not only in emergency settings, but also in routine anesthesia (e.g., prone position for lumbar spine surgery). Methods: The aim of the study was to compare the classic Macintosh blade laryngoscope with two videolaryngoscopes: the Pentax-AWS and the Intubrite in a simulated scenario of a manikin placed in a sitting and prone position. Additionally, intubation with the use of all three devices was performed in a standard supine position as the control group. The time of intubation and the pressure exerted on the tongue was assessed. The ANOVA Friedman (analysis of variance) and Wilcoxon with Bonferroni correction tests were used for statistical analysis. Results: The time of intubation in a prone position was significantly shorter for the Pentax-AWS videolaryngoscope compared to the Macintosh and the Intubrite. There were no significant differences in the obtained results of the evaluated devices in sitting and standard positions. The lowest pressure exerted on the tongue was with the Pentax-AWS, followed by the Intubrite and the Macintosh laryngoscopes. Conclusions: The use of the Pentax-AWS was associated with faster tracheal intubation, creating lower pressure on tongue when compared with standard Macintosh and Intubrite laryngoscopes in both prone and sitting positions.

## 1. Introduction

There is an ongoing discussion whether patients for general anesthesia in the prone position could be induced and intubated prone [1]. The management of an accidental extubation of a patient in the prone position requires an appropriate approach [2,3], with the need for adequate equipment. An unexpected extubation in a prone position may be a life-threatening situation [4,5] and therefore studies evaluating different airway devices should be conducted. The search for an ideal method and device is important to increase the safety of patients anesthetized in the prone position [5]. Intubation of a patient who is not positioned supine may be required not only in an emergency, but also in routine anesthesia (e.g., for lumbar spinal surgery [5,6]). 

Intubation in a non-standard position such as face-to-face is also used in anesthesia for patients with various medical conditions that do not allow for the patient to be placed in a supine position (e.g., rheumatoid arthritis [7]). In emergency settings, a face-to-face intubation may be utilized in trauma patients entrapped in crashed vehicles [8]. Therefore, the ability to intubate in different positions may be important in day-to-day clinical practice [9]. 

At present, there are many different devices and methods available for tracheal intubation. 

The assessment of intubation devices requires standard and repeatable conditions. Due to ethical issues and methodological problems, the manikin model of various different scenarios seems to be the most proper and acceptable.

The hypothesis of this study was that new airway management devices such as Intubrite and Pentax AWS videolaryngoscopes would provide faster and more efficient intubation in patients placed in a prone position. 

## 2. Materials and Methods

**Figure 1:**
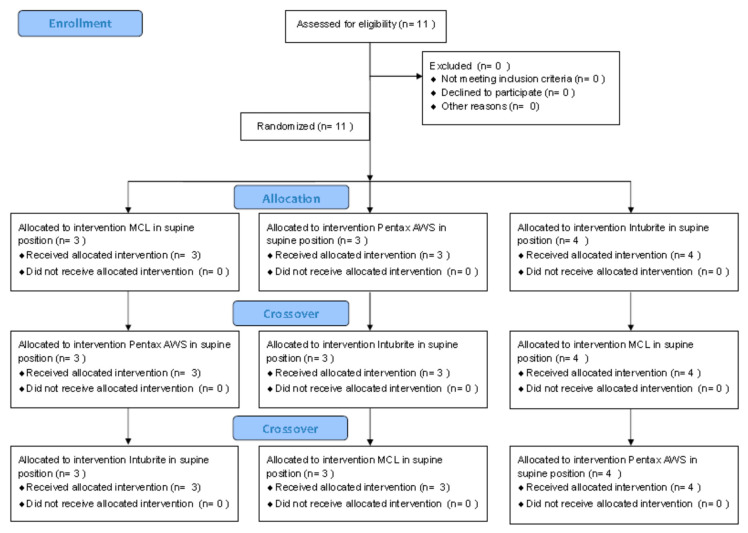

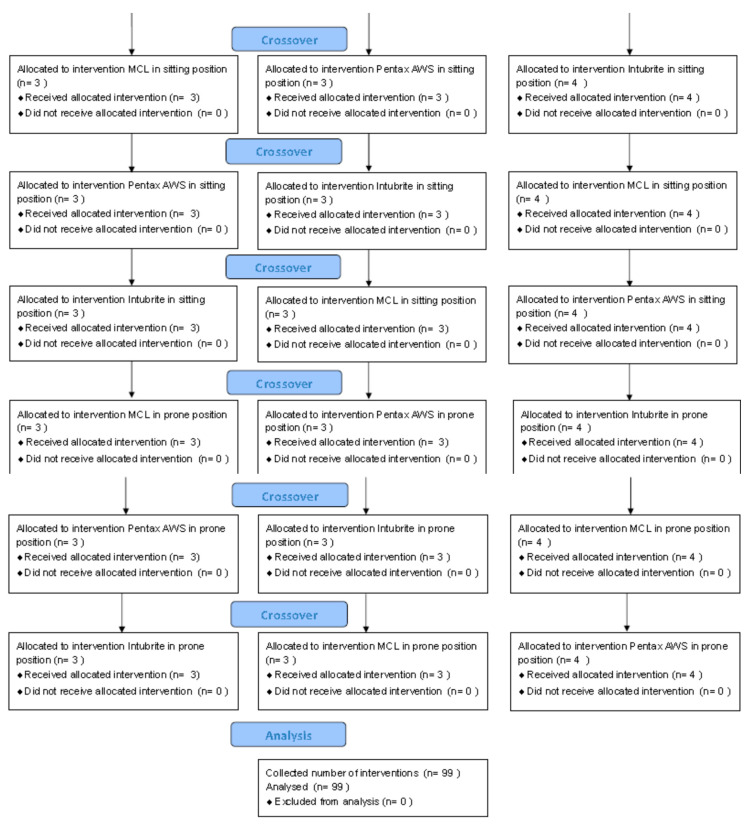
The CONSORT 2010 Flow Diagram.

**Figure 2:**
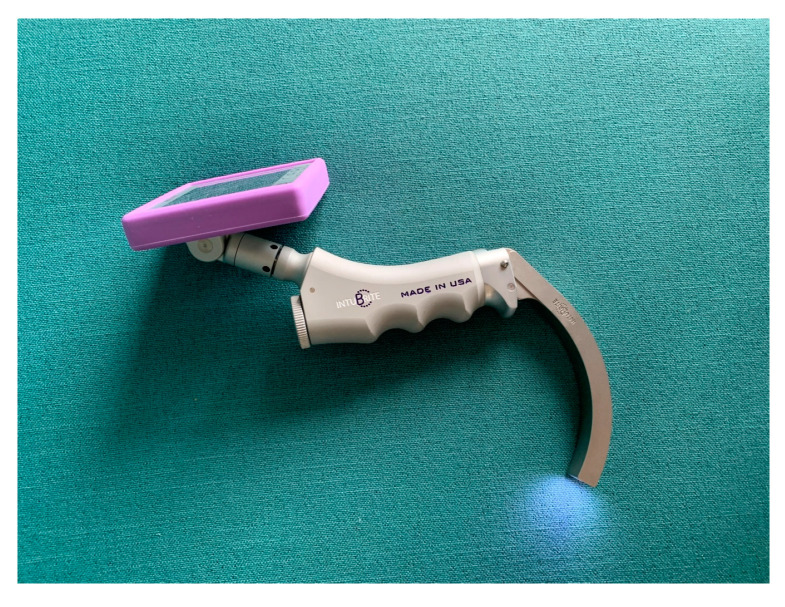
Intubrite videolaryngoscope (author’s own material).

**Figure 3:**
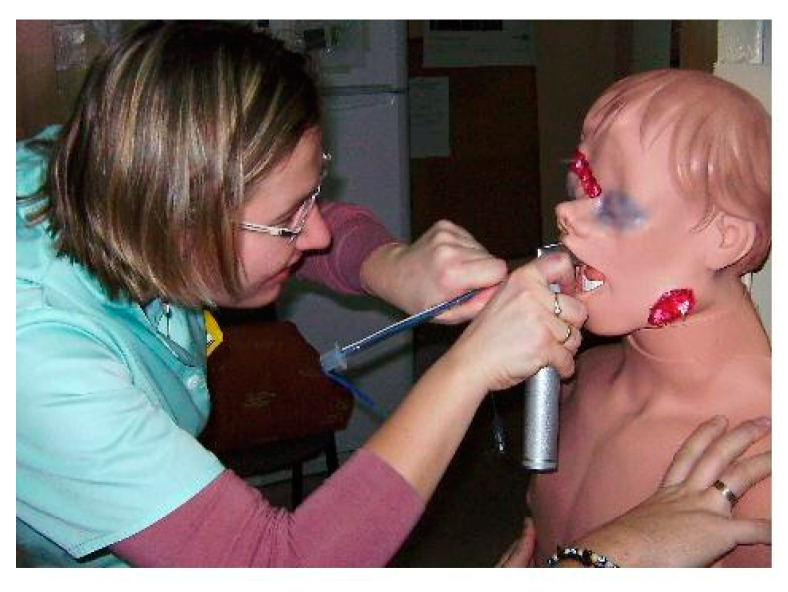
Intubation of a trauma manikin in a sitting position (Author’s own material).

**Figure 4:**
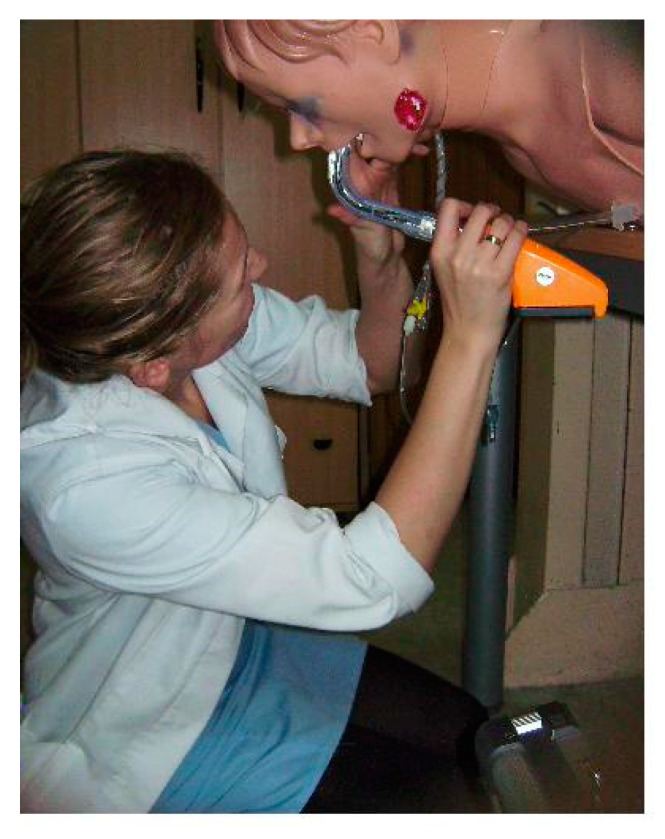
Intubation of a trauma manikin in a prone position (Author’s own material).

After consulting the Institutional Ethics Committee, specific ethical approval for this study was not considered to be necessary. The study was performed as part of the University Department’s scientific activity with institutional funds and was done at the University’s teaching hospital. This study comprised a randomized cross-over design. The CONSORT 2010 flow diagram is presented in Figure 1. The sequence in which each participant used the evaluated devices was randomized using a closed envelope method (e.g., (1) MCL, (2) Intubrite, (3) Pentax-AWS, and the randomized sequence was 1,3,2, or 3,1,2, or 2,3,1, etc.). In this study, we compared the efficacy and safety (a surrogate parameter of safety was the pressure on the tongue) of the classic Macintosh blade laryngoscope (MCL) and two modern videolaryngoscopes (i.e., the Intubrite (Intubrite LLC, Vista, CA, USA) (Figure 2) and the Pentax-AWS (Pentax, Tokyo, Japan) (Figure 4)) in a simulated scenario of a manikin placed in different positions. The three basic scenarios were created: with a manikin placed supine (no difficulties, control), in a sitting position (medium difficulties, Figure 3) and prone (significant difficulties, Figure 4).

The Trauma Intubation Head (Woodstock, NY, USA) manikin was used for the difficult intubation scenario in our study. In this model, there is a built-in system for mimicking tongue hematoma or edema. This system requires filling with different volumes of air according to the needs. For tongue pressure measurement during intubation, we connected this system to a manometer, after filling it with five cm^3^ of air. This direct measurement of pressure exerted during laryngoscopy by an operator using different devices enabled us to assess both the safety and difficulty of the performed procedure.

For the study, we recruited 11 experienced anesthetists (all specialists having at least six months of clinical experience after completing their specialist training). Demographic data are provided in Table 1. All participants had clinical experience in anesthesia and all worked at the Łódź Medical University teaching hospital. None of the participants had previously used either of the evaluated videolaryngoscopes. Participation was voluntary and all data anonymized. Each participant was given a standardized demonstration of the studied devices by one of the investigators before starting the study. This included an explanation and oral instructions on how to use them. The participants were then shown the vocal cords with each device and the recommended technique to successfully intubate the Trauma Intubation Head manikin. The participants were allowed to practice with each device until one successful intubation had been achieved in the normal (non-difficult) airway scenario. All intubations were performed with a size 7.0 mm cuffed Endo-Tracheal Tube (ETT, Sumi, Sulejowek, Poland) and the cuff was inflated and deflated with a 20 mL BD syringe (BD Drogheda, Ireland). The ETT was lubricated with Laerdal Airway Lubricant for training manikins before each intubation attempt. No time limit was set up during the intubation practice. All intubations were performed orally. In all scenarios, size 3 blades were used for all studied devices.

The primary endpoints were the time necessary for successful tracheal intubation, the number of successful intubation attempts, and pressure exerted during procedures. In each case, the efficacy of tracheal intubation was confirmed by manikin ventilation. A failed intubation attempt was defined as failure to place the ETT between the cords and/or an attempt requiring >120 s to perform. 

Timing commenced when the intubation device was grabbed by a participant. Time to successful intubation was defined as the time taken from grabbing the device until the end of one successful lung inflation using a self-inflating bag (Medline, Poland). After each intubation attempt, the final ETT position was verified by the investigator. Visualization of the vocal cords was evaluated using the Cormack–Lehane score. All data were recorded by the two unblinded investigators.

The statistical analysis of collected data was performed using STATISTICA ver. 10.0 software (Statsoft, Poland). ANOVA Friedman (analysis of variance, repeated measures, nonparametric, adjustments for multiple comparisons) and post-hoc Wilcoxon tests for paired systems with Bonferroni correction were used. There was no power analysis before study, however, with the estimation for paired systems for 11 measurements and a level of significance of 0.017 (0.05/3 comparisons) and 80% of power, it should be possible to detect a difference at the level of 1.13 of standard deviation. A *p* value < 0.05 was considered statistically significant. Sample size was not calculated before study. This trial was explorative.

## 3. Results

**Figure 5:**
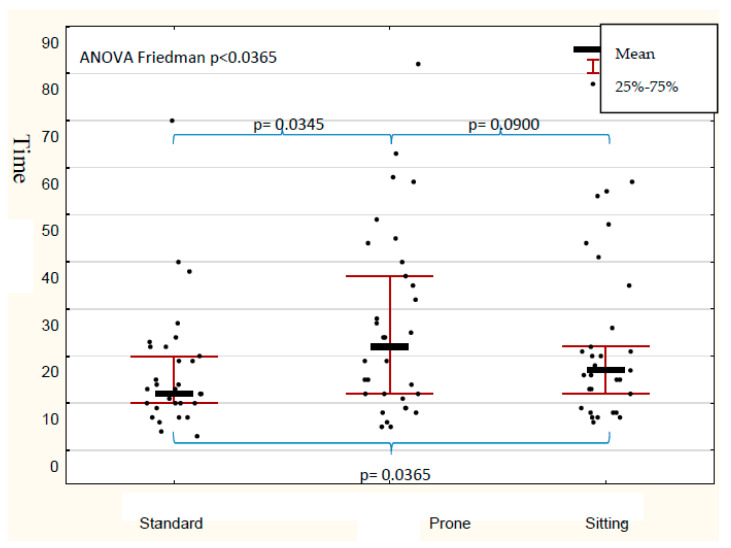
Time of intubation with all devices in different positions of the manikin [s].

The mean time necessary for successful intubation in the prone position was significantly longer in comparison with a standard position for each device (*p* = 0.0356, Figure 5) except for Pentax-AWS (Table 2). The longest intubation time was noted when the MCL was used. However, this was only observed in the prone position. In other positions, the intubation time was comparable (*p* > 0.05). The influence of the used device on the mean time of intubation during all attempts was not statistically significant (*p* = 0.1948). The post-hoc test revealed that the time of intubation in the prone position was significantly longer when compared to the standard, independently of the used device (*p* = 0.0345). The difference between sitting and prone was on the border of significance (*p* = 0.0900). The time to intubation in the prone position was shorter for the Pentax-AWS videolaryngoscope when compared with the Macintosh and Intubrite (Table 2), but only a significant difference between the Macintosh and Pentax-AWS laryngoscopes (Table 2). In the case of Intubrite and Pentax-AWS, there was no significant difference in the time of intubation (*p* = 0.2102).

**Figure 6:**
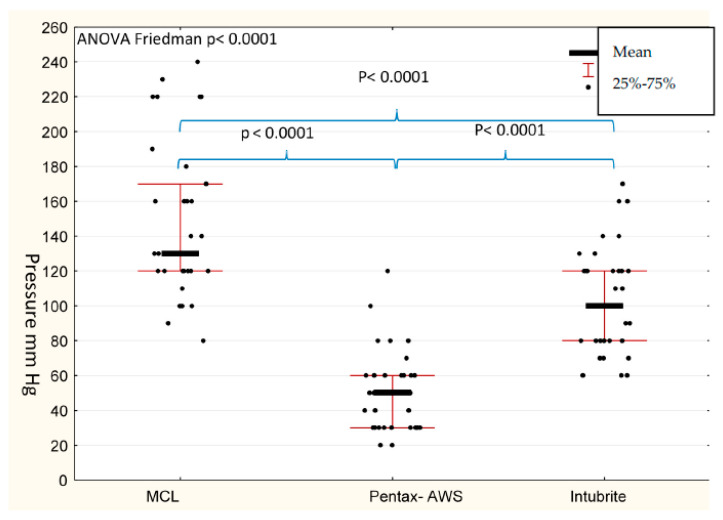
Pressure exerted with the use of different devices in all positions [mmHg].

When analyzing pressure created on the tongue during intubation efforts, there was no significant influence of position on created pressure (*p* = 0.7934). However, the mean value of the pressure exerted on the tongue by operators during tracheal intubation in all attempts was significantly dependent on the type of device used (*p* < 0.0001) (Table 3). The lowest value was recorded with the use of the Pentax-AWS device, followed by the Intubrite. The highest pressure was noted when the classic Macintosh blade laryngoscope was used (Table 3). The pressure exerted on the tongue was the highest for MCL and the lowest for Pentax AWS in all positions with statistical significance (Table 3). The pressure exerted was the highest for all devices in the prone position, but only Pentax-AWS presented significantly lower on tongue pressure than MCL and Intubrite (Table 3). The difference between Intubrite and MCL in the prone position was not statistically significant (Table 3). The difference between Pentax AWS and Intubrite videolaryngoscopes was significant in all positions with *p* = 0.003, *p* = 0.0071, *p* = 0.0018 in the supine, prone, and sitting positions, respectively. The Pentax AWS exerted the lowest pressure in all positions (Figure 6). 

**Figure 7:**
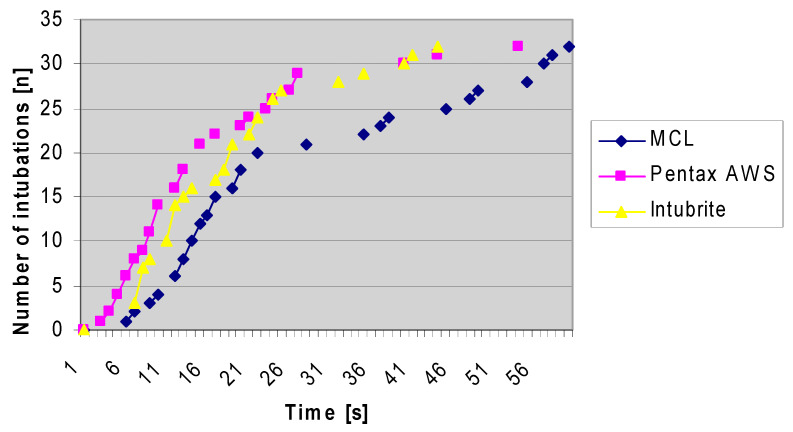
Cumulative success rate related to time [n of intubations].

The intubation success rate was 100% for all videolaryngoscopes in the first attempt. The cumulative success rate (time-related) was the highest for the Pentax-AWS and the lowest for the MCL (Figure 7).

The Cormack–Lehane (CL) score was 1 for all videolaryngoscopy attempts. For the MCL, the mean CL score was 2.1 ± 0.83 in the standard, 3.2 ± 0.95 in the prone, and 2.5 ± 0.74 in the sitting positions. 

## 4. Discussion

### 4.1. Significance of the Study Results

This study confirmed that the use of videolaryngoscopes allowed for the time necessary to intubate a patient in a non-standard position-prone to significantly shorten. This may be especially important for anesthesia in patients in the prone position (for example, lumbar spinal surgery). The results of our study can add information that can be useful for clinical practice regarding the choice of a device for intubation in non-standard positions (e.g., the choice between channeled or non-channeled videolaryngoscopes).

### 4.2. Comparison with Other Published Studies on This Topic-Similarities and Differences

In many published studies with the use of manikin models, the mean time for tracheal intubation was shorter when videolaryngoscopes were used [10]. In our study, we observed such as difference in a high difficulty prone position. Lack of the time differences in the standard and sitting positions may result from the fact that we recruited experienced anesthetists to conduct the procedures during our study. 

There are several studies available on the device that we tested (i.e., the Pentax-AWS). In a study comparing the time of intubation with the use of the Pentax–AWS by experienced and inexperienced personnel in an emergency unit, investigators identified longer times necessary for novices in order to achieve a successful intubation [10]. More importantly, the time necessary for inexperienced users to perform the effective airway management with the use of this device was still short, so they concluded that the Pentax-AWS may be a suitable device particularly for less experienced personnel such as novice advanced life support providers [11]. 

More importantly, we observed that laryngoscopy with the Pentax-AWS device was associated with significantly lower pressures exerted on the tongue during tracheal intubation. This indicated that the use of an objective measurement was much easier and provided safer intubation when compared with the other two devices. Additionally, the difference in the mean pressure exerted on the tongue during intubation was significant even between the Intubrite and the Macintosh blade laryngoscope. These results are similar with those achieved in previous studies [12,13,14]. 

In a prospective manikin study, Ong et al. compared indirect intubating devices: the Pentax-AWS, Truview Evo2, and Clarus Levitan fiberoptic stylet with a direct Macintosh laryngoscope in both normal and difficult airway scenarios. They observed shorter intubation times with the Pentax-AWS device. In the normal airway scenario, the performance of intubation evaluated by operators in the visual analogue scale (VAS) was easier with the use of the AWS and Macintosh laryngoscopes. In the difficult airway scenario, the VAS score of the AWS was lower than the other three devices. Additionally, the glottic view (Cormack–Lehane score) was better for the AWS and other indirect devices than for the Macintosh laryngoscope. The authors concluded that the AWS device had the best performance in a normal and difficult airway scenario [15].

Another important piece of information from our study is that, although both tested video laryngoscopes offered easier and safer tracheal intubation in comparison with a classic Macintosh blade laryngoscope, there was still a significant difference even between them. It is especially important because of the growing number of different video devices introduced into clinical practice. Therefore, it may be easier to interpret confusing observations and results of the performance of video devices [16]. Both videolaryngoscopes (i.e., the Pentax-AWS and Intubrite) also differ in construction and operation. The Pentax-AWS belongs to a group of laryngoscopes with a channel for a tracheal tube (Figure 2). The Intubrite device has a handle and blade similar to a standard laryngoscope and a video display monitor is attached to the handle (Figure 1). The differences in construction and operation of these video devices have influenced the results of the study. It seems that devices with a channel for a tube are more handy when used in an entrapped patient scenario.

In our manikin study, the use of the Pentax-AWS videolaryngoscope significantly shortened the time necessary for intubation in the prone position when compared to both the Macintosh and the Intubrite videolaryngoscopes. The pressure exerted on the tongue by the Pentax-AWS had the lowest value in all positions, so the risk of intubation-induced trauma complications should be lower when compared with the other devices in this study. At the same time, the lowest value of this pressure indicated the easiest method of tracheal intubation. 

In our study, as expected, we observed that the mean time required for successful intubation in the prone position was significantly longer when compared with a standard supine position for each device. Komasawa et al. performed a study that evaluated the performance of the Pentax-AWS for tracheal intubation in a manikin placed in the supine, left-lateral decubitus, right-lateral decubitus, prone, and sitting positions [17] but did not compare this device to other devices. They found that tracheal intubations with AWS in all of the five above-mentioned positions were successful, but the intubation with the patient sitting, right-LT, and prone positions was more difficult and required more time than when the manikin was placed supine. 

There is limited clinical data on the use of videolaryngoscopes in the sitting or prone positions. Intubation in the prone position is often required in emergency settings with the intubating laryngeal mask airway (ILMA) [18] or as routine anesthesia practice for patients with chronic low back pain who are candidates for lumbar fusion operations [19]. The success rate depends on the experience of the anesthetist and may reach 98.8% in centers where this procedure is performed very often [19]. Intubation in the prone position for elective surgery should be performed using the awake fiber-optic technique [20]. There is only one case report in the current literature on the use of videolaryngoscope, in this case the Pentax AWS, for awake prone intubation in morbidly obese patients scheduled for discectomy for lumbar disc herniation [21]. Intubation in a sitting position with videolaryngoscopes was described in two case reports. One case report was on intubation in a semi-sitting position of a morbidly obese patient using the Pentax AWS [22]. The second case was an adult patient with acute epiglottitis managed using the Storz V-Mac device [23]. 

There is an ongoing debate whether general anesthesia for patients operated on in the prone position (e.g., lumbar spine surgery may be performed using laryngeal mask airways [24,25,26]). Several studies have shown that laryngeal masks are effective for airway management in patients placed in a prone position [4,27,28,29,30,31,32,33]. There is no doubt that in the sudden extubation of a patient in a prone position, laryngeal masks are a useful tool [30,31,32,33]. However, many clinicians believe that for a patient’s safety, tracheal intubation is necessary in case of placing him/her in a prone position [26,34]. Videolaryngoscopes are often used successfully for intubation in a prone position [35]. Therefore, we believe that our study carries some useful data and may give some clues in the case of emergency intubation in a prone position (e.g., unexpected extubation in patients having lumbar spine surgery or for elective use of videolaryngoscopes in awake videolaryngoscopy-assisted intubation in prone or sitting positions [36]). Studies comparing different videolaryngoscopes in patients with difficult intubation have confirmed that there are important differences in the performance of various different devices [37]. Van Zundert noted in his review that airway management in patients placed in non-supine positions requires experience and training of such techniques [9]. Emergency intubation of patients who cannot lay flat due to different conditions requires special skills and equipment in order to be performed safely [38]. Therefore, we believe that studies comparing video intubating devices are necessary to evaluate both their safety and efficacy.

### 4.3. Limitations of the Study

This study has several limitations. First of all, it was a manikin study. Due to technical and ethical issues, it may be difficult to perform a study involving human subjects. Although manikin studies have their disadvantages (e.g., no stress factor, controlled and stable environmental conditions, difference in chest and other structures of manikin compliance compared to real tissue), they are still valuable as they suggest which approach may be more beneficial to real patients. The manikin model of intubation should offer the same conditions for all performed procedures, creating a comparable basis for different emergency scenarios. The limitations of this model are the lack of other pathophysiological disturbances, anatomical difficulties, and unpredictable reactions. Second, the number of participants was limited due to the inability to recruit more people at the time the study was conducted. A sample size calculation was not conducted and the sample was a convenience sample. Sample size analysis was performed post-hoc. As results of our study showed that tracheal intubation using Pentax AWS was significantly faster in the prone position by 23 s (compared to MCL) and 12 s (compared to Intubrite), we assumed that a 17-s difference in intubation time was significant in this study. Based on a measured maximum SD of 22 seconds for intubation time in this study, a sample size of eight in each group would have 90% power to detect a 17-s difference in intubation times between the three groups, assuming α = 0.05. Eleven participants recruited in this study secured a safety margin. Finally, another potential limitation of the study is that all the observations were carried out by a single unblinded researcher. Despite these limitations, we believe that the results of our work may add some important information to the current literature related to emergency airway management.

Strengths of this study:

1. This is the first study that compares two types of videolaryngoscopes, channeled and non-channeled, in relation to the effectiveness and possible complications of tracheal intubation in specific patient positioning in both the sitting and prone positions.
2. New techniques and devices for airway management should be tested for possible application in very difficult airway scenarios (e.g., in entrapped patients or for intubation in patients requiring prone position such as for spinal neurosurgery procedures). This study provides answers on the efficacy of the evaluated videolaryngoscopes in such situations.
3. Although main limitation of this study is that it was a manikin study, the human study can be performed as a follow up of this study, taking into consideration the important results of this work.

### 4.4. Potential Implications for Future Research

Future research should concentrate on the evaluation of the use of videolaryngoscopes in patients in the prone position in clinical settings. Manikin studies proved that videolaryngoscopes are useful in such conditions and these studies showed that channeled videolaryngoscopes are more efficient than non-channeled ones in emergency situations such as during accidental extubation in patients in the prone position [39]. Studies in clinical settings are required to confirm such findings. 

## 5. Conclusions

Among the evaluated videolaryngoscopes, the Pentax-AWS in a simulated scenario of a patient placed in different positions offered faster tracheal intubation and created lower pressure on the tongue in comparison with the Intubrite and classic Macintosh blade laryngoscopes. 

## Figures and Tables

**Table 1:** Demographic data of participants. ETI—endotracheal intubations.

Gender	Experience Distribution	Estimated Number of Annual ETI Experience
7 female/4 male	Mean 4 years (6 months–6 years)	892 ± 78

**Table 2:** Time to successful intubation in simulated different positions [s] (Min-minimum, Max-maximum, SD-standard deviation). MCL-Macintosh Laryngoscope, * *p* value when compared with the MCL (control group).

Scenario	Laryngoscope	Min.	Max.	Mean	SD	*p* Value
Supine	MCL	6	70	13	18.93	-
	Pentax-AWS	3	40	12	10.98	0.2765 *
	Intubrite	7	24	12	5.75	0.2011 *
Prone	MCL	12	58	35	16.58	-
	Pentax-AWS	5	82	12	22.13	0.0422 *
	Intubrite	8	63	24	17.45	0.1365 *
Sitting position	MCL	13	57	17	17.33	-
	Pentax-AWS	6	54	15	15.9	0.1708 *
	Intubrite	7	41	17	11.32	0.0900 *

**Table 3:** Pressure created on the tongue during intubation in simulated different positions [mmHg]. (Min-minimum, Max-maximum, SD-standard deviation). MCL-Macintosh Laryngoscope, * *p* value when compared with the MCL (control group).

Scenario	Laryngoscope	Min.	Max.	Mean	SD	*p* Value
Supine	MCL	100	220	120	46.27	-
	Pentax-AWS	20	100	40	28.57	0.0006 *
	Intubrite	60	170	100	22.52	0.0139 *
Prone	MCL	80	230	130	44.78	-
	Pentax-AWS	20	60	50	23.66	0.0009 *
	Intubrite	70	160	120	37.24	0.0547
Sitting position	MCL	90	240	130	46.74	-
	Pentax-AWS	30	120	60	14.89	0.0057 *
	Intubrite	60	130	100	29.41	0.0010 *
